# Prediction of stable radon fluoride molecules and geometry optimization using first-principles calculations

**DOI:** 10.1038/s41598-023-29313-5

**Published:** 2023-02-18

**Authors:** Jaeeun Kang, Ina Park, Ji Hoon Shim, Duck Young Kim, Wooyong Um

**Affiliations:** 1 Division of Advanced Nuclear Engineering (DANE), Pohang University of Science and Technology (POSTECH), 77 Cheongam–ro, Nam–Gu, Pohang, Gyeongbuk 790–784 Republic of Korea; 2 Department of Chemistry, Pohang University of Science and Technology (POSTECH), 77 Cheongam–ro, Nam–Gu, Pohang, Gyeongbuk 790–784 Republic of Korea; 3 Center for High Pressure Science & Technology Advanced Research, Shanghai, People’s Republic of China; 4 Division of Environmental Sciences and Engineering (DESE), Pohang University of Science and Technology (POSTECH), 77 Chongam–ro, Nam–Gu, Pohang, 790–784 Republic of Korea; 5 Nuclear Environmental Technology Institute (NETI), Pohang University of Science and Technology (POSTECH), Pohang, Gyeongbuk 790–784 Republic of Korea

**Keywords:** Environmental sciences, Natural hazards

## Abstract

Noble gases possess extremely low reactivity because their valence shells are closed. However, previous studies have suggested that these gases can form molecules when they combine with other elements with high electron affinity, such as fluorine. Radon is a naturally occurring radioactive noble gas, and the formation of radon-fluorine molecules is of significant interest owing to its potential application in future technologies that address environmental radioactivity. Nevertheless, because all isotopes of radon are radioactive and the longest radon half-life is only 3.82 days, experiments on radon chemistry have been limited. Here, we study the formation of radon molecules using first-principles calculations; additionally, possible compositions of radon fluorides are predicted using a crystal structure prediction approach. Similar to xenon fluorides, di-, tetra-, and hexafluorides are found to be stabilized. Coupled-cluster calculations reveal that RnF_6_ stabilizes with O_h_ point symmetry, unlike XeF_6_ with C_3v_ symmetry. Moreover, we provide the vibrational spectra of our predicted radon fluorides as a reference. The molecular stability of radon di-, tetra-, and hexafluoride obtained through calculations may lead to advances in radon chemistry.

## Introduction

A noble gas with a fully filled outer shell is not reactive. When argon was first discovered, chemists found that it was not reactive with the other elements in the periodic table, and noble gases were generally considered to be unreactive. In the 1930s, Pauling^1^ predicted that xenon (Xe) would be able to form compounds with fluorine. In related experiments, they succeeded only in corroding the walls of a quartz flask and were unaware of the presence of new compounds in it^2^. After several trials, xenon and fluorine readily reacted and formed the solid XeF_4_, which is stable even at room temperature^3^. The structures of xenon difluoride (XeF_2_) and xenon tetrafluoride (XeF_4_) were identified using their vibrational spectra^4^. However, the structure of xenon hexafluoride (XeF_6_) is controversial regarding the stereo activity of valence electron lone pairs^5^. Experimental evidence obtained using electron diffraction^6^ and vibrational spectroscopy^7^ suggests that XeF_6_ forms a distorted octahedral symmetry^8^.

Radon (Rn) is a noble gas like Xe; it is a naturally occurring radioactive material (NORM) found in the underground environment. It has a high density (9.73 g/L at standard temperature and pressure) and solubility (230 cm^3^/L at 20 °C)^9^ among the noble gases. When gaseous Rn is inhaled, it either emits alpha rays directly or decays into daughter radionuclides, which may cause lung cancer^10^. In addition, owing to its short half-life (only 3.82 d), experiments and studies involving radon have been limited thus far. Heavy noble gases, such as Xe and Rn, are both rare elements and highly radioactive gases; therefore, they present various challenges to experimental researchers. Radon difluoride (RnF_2_) was synthesized by Fields^11^; it formed nonvolatile RnF_2_ when exposed to fluorine and heated to 400 °C. RnF_2_ is currently the only known radon molecule. Owing to high temperature and pressure requirements, obtaining a complete solid RnF_2_ sample in a natural environment is challenging.

Computational studies based on first-principles density functional theory (DFT) are regarded as a practical alternative for the study of these gases as DFT has been successfully applied for the detailed analysis of a vast number of materials. The DFT approach can be used to provide qualitative predictions of geometric features and determine various chemical and physical properties. However, computational studies based on first-principles about interactions of Rn atoms with environments (e. g., Rn·H_2_O complex)^12^ or formation of Rn molecules are very rare.

A previous study^13^ used redundancy analysis to determine the Rn content in groundwater, and they found a positive correlation with fluorine concentration. This raised the questions of whether Rn can interact with fluorine, and how the radon fluoride reaction should be elucidated. Only a few theoretical studies^14^ have demonstrated the relationship between Rn and fluorine, but most of this literature is focused on Xe or Kr, and Rn was not treated as the main subject. Malli^15^ and Filatov^16^ suggested the atomization energy and RnF bond length of radon hexafluoride (RnF_6_) at the Hartree–Fock (HF) and MP2 levels of theory. However, they assumed only octahedral RnF_6_ geometry as the initial structure and did not consider other possibilities. Our study aims to understand the possible chemical bonding between Rn and fluorine and its geometry through first-principles calculations. Additionally, the vibrational frequencies of radon di-, tetra-, and hexafluorides were calculated at the coupled-cluster level, suggesting as a reference. Herein, first-principles calculations were performed for Xe and Rn fluorides with various basis sets at different methods to compare geometrical parameters. Subsequently, the most stable combination describing the Xe and Rn fluorides was provided for geometry optimizations.

## Results

### Xenon hexafluoride (XeF_6_)

Gillespie^17^ and Hedberg^18^ predicted that the geometry of ground state XeF_6_ is a distorted octahedron with C_3v_ point symmetry. According to prior studies, two characteristic bond lengths of Xe-F are experimentally determined to be 1.85 and 1.94 ± 0.036 Å^19^, and the∠F-Xe-F of 114.9° and 81.0° were obtained at the self-consistent field (SCF) level^20^. To calculate the geometries of XeF_6_, initial symmetries were set to C_2v_, C_3v,_ and octahedral (O_h_); subsequently, the bond lengths and angles were fully relaxed. These three symmetries of XeF_6_ were calculated using the DFT, Møller–Plesset second-order perturbation theory (MP2)^21^, and coupled cluster singles and doubles (CCSD)^22–25^ methods. The calculated structures by DFT and MP2 are described in Supplementary Tables S1 and S2 of the Supplementary Information (SI).

Certain calculated structures converged equivalently to the O_h_ molecular geometry, even though they started at different initial point groups such as C_2v_ or C_3v_, according to the DFT and MP2 results. Other calculated structures stay at their initial symmetry and they tended to slightly overestimate the bond length compared with the experimentally obtained values by ~ 0.06 Å. Note that bond lengths determined using the MP2 method were rather closer to the experimental values compared with those provided by the DFT method. The angles obtained from these two methods tend to be underestimated compared with other calculated values, and the C_3v_ structure was stable at the lowest energies for all basis sets, except in the case of the O_h_-converged structure. The energy differences for other symmetries compared with that of C_3v_ are denoted as ΔE. The bond angles calculated using the DFT and MP2 methods are deviated noticeably from other calculated values up to 9.55%.

In case DFT and MP2 gave the same answer, we additionally applied CC method as a cross-check to validate our calculation results. The XeF_6_ structure was determined at the CCSD level, as presented in Supplementary Table S3. At the CCSD level, the relativistic effect of Xe was not considered, and three initial symmetries were the same as those in the DFT and MP2 results. The initial symmetries were maintained during the relaxation and C_3v_ symmetry is predicted to be the ground state for all basis sets. For the LANL2DZ and CEP-31G basis sets, the calculated lengths have a good agreement with experimental data, with a small difference of the order of two decimal places in angstrom. The angles for the C_3v_ symmetry were different from other calculated values only by approximately 1° for these basis sets.

In summary, the bond lengths and bond angles determined using the DFT and MP2 methods were overestimated and underestimated respectively, implying stabilizing the metastable stereo-isomers was challenging. When it comes to XeF_6_, CCSD level calculations with non-relativistic effect gave quantitative agreement with experimental data, which is consistent with several previous studies^26,27^.

In the case of many-electron atoms, the relativistic contraction of inner-shell orbitals by screening affects the outer-shell orbitals^28^. This may result in a significant impact on the chemical and physical properties of heavy inert gases in the lower half of the periodic table^29^. Therefore, the relativistic effect of Xe was further considered at the CCSD level using the DKH Hamiltonian. Supplementary Table S4 summarizes the bond length and total energies of XeF_6_ for the three geometries with DK3 basis sets. The relativistic effect did not significantly affect geometric parameters. The C_3v_ structure possesses lower total energy than the O_h_ structure and by C_2v_ structure by 18.88 kcal/mol and 8.28 kcal/mol, respectively using the cc-pVTZ-DK3 basis set. For aug-cc-VTZ-DK3, the overall energy differences, $\Delta \mathrm{E}$ were lower than those of cc-VTZ-DK3, although there was a tendency to overestimate the bond length by approximately 2.06%. In the DKH calculation, the angle was reduced by approximately 5º compared with the case employing the LANL2DZ basis set in the NR calculation. Although the NR calculation produced values that were in better agreement with the experimental results for XeF_6_, the DKH calculation also exhibited small differences that were within 0.01 Å. Even when relativistic effects were not considered, the CCSD level was found to be consistent with the experiment for XeF_6_ (C_3v_). Our calculation of XeF_6_ parameters can be used for the following computational study to determine the effective computational level depending on initial symmetry in Rn-F chemistry.

### Radon difluoride (RnF_2_) and radon tetrafluoride (RnF_4_)

The atomization energies of argon, krypton, and xenon difluoride indicate that these molecules tend to become stable with increasing atomic number^30^. Following this trend, radon difluoride is expected to be more stable than the corresponding xenon molecules^31^. Therefore, the simpler structures of radon difluoride (RnF_2_) and radon tetrafluoride (RnF_4_) were first determined. However, the only reported experimental values for radon molecules are those for RnF_2_, which were proposed by Fields^11^. Therefore, first, the stability was investigated by calculating the formation energies of radon fluorides. The convex hull diagram in Supplementary Fig. S1 indicated that the lowest energy can occur between the Rn and binary F_2_ phases. The energy convex hull graph helps to understand that RnF_6_ is the most stable molecule along the thermodynamic route while RnF_2_ and RnF_4_ may exist as metastable forms. Speculatively, as there is an experimental observation of RnF_2_, we can easily speculate the possible formation of RnF_4_ and RnF_6_ as well. For structural optimization, the geometry of RnF_4_ and RnF_6_ were determined by CCSD, with relativistic effects (DKH). As presented in Table 1, the bond lengths of RnF_2_ and RnF_4_ were estimated to be 2.04–2.05 Å and 2.00–2.01 Å, respectively, depending on the basis sets employed. The optimized structures of RnF_2_ and RnF_4_ were obtained as linear and square planar, as in the case of xenon fluoride, respectively. The bond lengths of radon fluorides were longer than those of xenon fluorides, which is a predictable result considering the larger atomic size of radon. In addition, radon di- and tetrafluoride retained a similar structure to xenon fluorides.

**Table 1:** Geometric parameters were determined with relativistic effect by CCSD of RnF_2_ and RnF_4_.

Molecules	Basis set	Bond length (Å)	Bond angle (º)	Energy (Hartree)
RnF_2_	aug-cc-pVTZ-DK3A	2.05	180	− 483.13
	cc-pVTZ-DK3A	2.04	180	− 483.07
RnF_4_	aug-cc-pVTZ-DK3A	2.01	90	− 682.24
	cc-pVTZ-DK3A	2.00	90	− 682.17

### Radon hexafluoride (RnF_6_)

The symmetrically optimized structures and energies for C_2v_, C_3v,_ and O_h_ obtained using DFT are summarized in Supplementary Table S5. Several previous studies^15,16,20^ predicted the bond lengths of RnF_6_, but they exhibited large differences in bond length depending on the type of calculation employed. For the DFT simulations, all basis sets converged equivalently to O_h_, and the bond lengths of RnF_6_ differed by up to 0.09 Å compared with the experimental XeF_6_ structures^19^. In general, the stronger the attraction between bonding atoms, the shorter the bond, and the greater the atom size, the longer the bond. As expected, the bond lengths of RnF_6_ were greater than those of xenon, although the difference between these was only 0.09 Å. As Rn has an atomic radius that is approximately 0.12 Å larger than that of Xe, the calculated bond lengths for radon fluoride were predicted to be shorter, as expected. Using the enthalpy of RnF_2_ formation^14,32^, Rn was theoretically demonstrated to form relatively strong covalent bonds compared with other noble gases^33^. In that study, Rn was expected to form shorter and more stable bonds with fluorine than XeF_6_, and these results are consistent with our calculations.

The calculated structures and relative energies for RnF_6_ for the MP2 method are described in Supplementary Table S6. For all converged O_h_ structures, the RnF_6_ bond length varied between 1.98 and 2.03 Å, and these values tended to be slightly underestimated compared to the DFT results.

**Figure 1:**
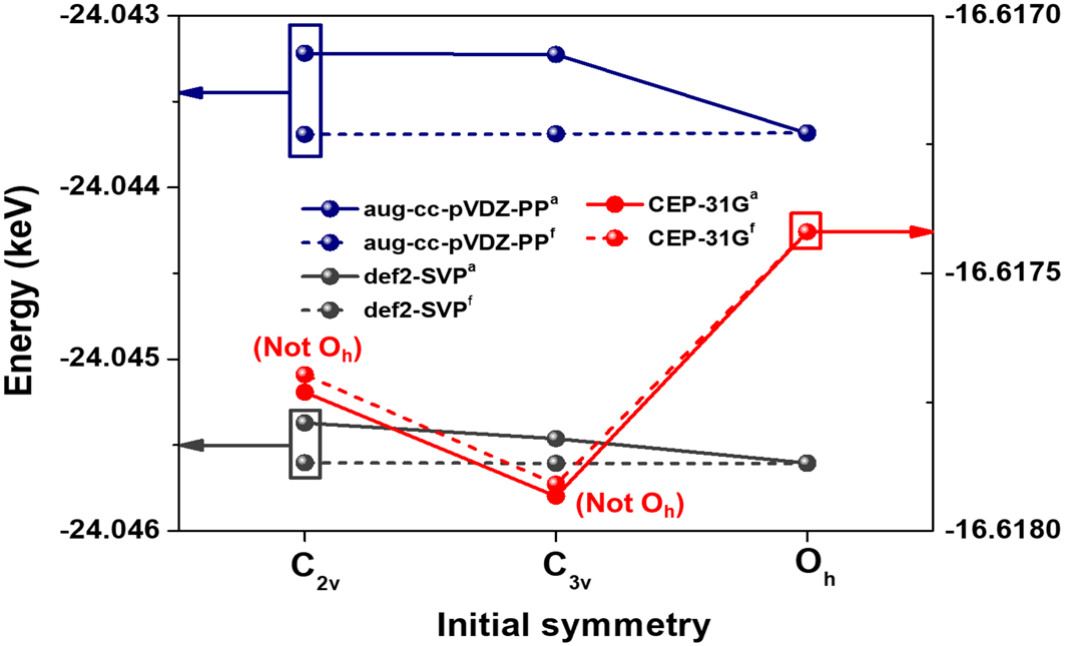
Comparison of the total energy (keV) for RnF_6_ calculated by the CCSD method. ^a^anglefix is the fixed given angles and ^f^ symmfollow is the freely relaxed bond length.

Unlike XeF_6_, all converged geometries exhibited O_h_ symmetry in the case of RnF_6_, irrespective of initial symmetry. Although stabilization of the metastable C_2v_ and C_3v_ state was attempted, this was only possible using a fixed bond angle, as shown in Fig. 1. These results reveal that the energy barrier between the O_h_ ground state and the other metastable states (C_3v_ and C_2v_) was particularly small, and therefore, the initial states easily relaxed into the O_h_ symmetry state. The bond lengths and total energies that were fully relaxed at the CCSD level are presented in Table 2. The radon fluoride bond lengths obtained using CCSD were slightly shorter for all basis sets compared with the other two methods (DFT and MP2). Unlike the DFT and MP2 results, the CEP-31G basis set was used to predict that C_3v_ symmetry was the most stable structure at the CCSD level, and this differed from the second most stable structure of C_2v_ by approximately 5 kcal/mol.

**Table 2:** Geometric parameters determined by CCSD for C_2v_, C_3v_, and O_h_ structures of RnF_6_.

Basis set	Initial symmetry	Bond length (Å)	Bond angle (º)	Energy (Hartree)	ΔE (kcal/mol)
aug-cc-pVDZ	C_2v_	a, b, c = 2.01	α = 89.98	− 883.58	O_h_
			β = 90.02		
	C_3v_	a, b = 2.01	α = 89.98		
			β = 90.02		
	O_h_	r = 2.01	θ = 90		
def2-SVP	C_2v_	a, b, c = 1.97	α = 89.91	− 883.65	O_h_
			β = 90.08		
	C_3v_	a, b = 1.97	α = 89.96		
			β = 90.00		
	O_h_	r = 1.97	θ = 90		
CEP-31G	C_2v_	a = 2.00	α = 115.86 α = 115.86	− 610.68	− 4.89
		b = 1.96			
		c = 1.93			
	C_3v_	a = 1.99	α = 110.22	− 610.69	
		b = 1.92	β = 80.72		
	O_h_	r = 1.97	θ = 90	− 610.67	− 11.29

Except for CEP–31G, other basis sets were converged equivalently to O_h_.

Additionally, DKH calculations were conducted considering the relativistic effect on the structural parameters of Rn. Table 3 summarizes the bond length and total energies of RnF_6_ for the three initial geometries (C_2v_, C_3v_, and O_h_). All initial geometries with different basis sets were converged to O_h_, and the bond lengths were estimated to be approximately 1.97 Å. Note that the relativistic effect on heavy atoms destabilizes the *d* and *f* orbitals because the inner (core) orbitals of the atom are strongly attracted to the nucleus. However, the outer orbitals are subject to minimal relativistic effects because the effective nuclear charge felt by the electrons decreases due to the screening effect. This usually causes the *s* and *p* orbitals to contract further and the *d* and *f* orbitals to expand, thereby stabilizing the 6*p* orbitals slightly but the 6*s* orbital strongly for Rn^16^. Therefore, this effect reduces the covalent radius of Rn and consequently induces a shorter radon fluoride bond length^16^, following the typical trend of bond shrinkage in molecules^15^. The relativistic effect leads to the presence of shorter bond lengths because of the stabilization of Rn atom inner orbitals.

**Table 3:** Geometric parameters were determined with relativistic effect by CCSD for C_2v_, C_3v_, and O_h_ structures of RnF_6_.

Basis set	Initial symmetry	Bond length (Å)	Energy (Hartree)	Converged symmetry
aug-cc-pVTZ-DK3A	C_2v_	a, b, c = 1.98	− 881.36	O_h_
	C_3v_	a, b = 1.98		
	O_h_	r = 1.98		
cc-pVTZ-DK3A	C_2v_	a, b, c = 1.97	− 881.29	O_h_
	C_3v_	a, b = 1.97		
	O_h_	r = 1.97		

**Figure 2:**
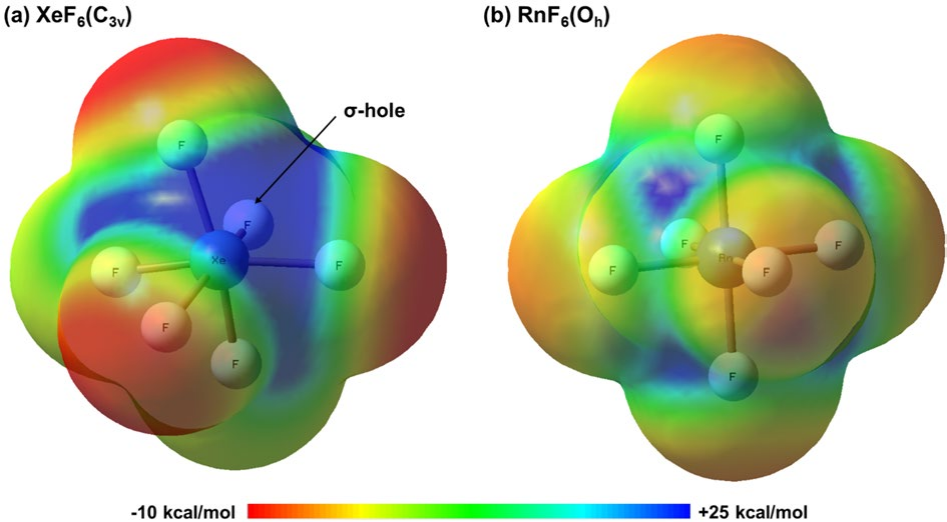
Molecular electrostatic potential (MEP) surface mapped (**a**) XeF_6_ (C_3v_) and (**b**) RnF_6_ (O_h_).

**Figure 3:**
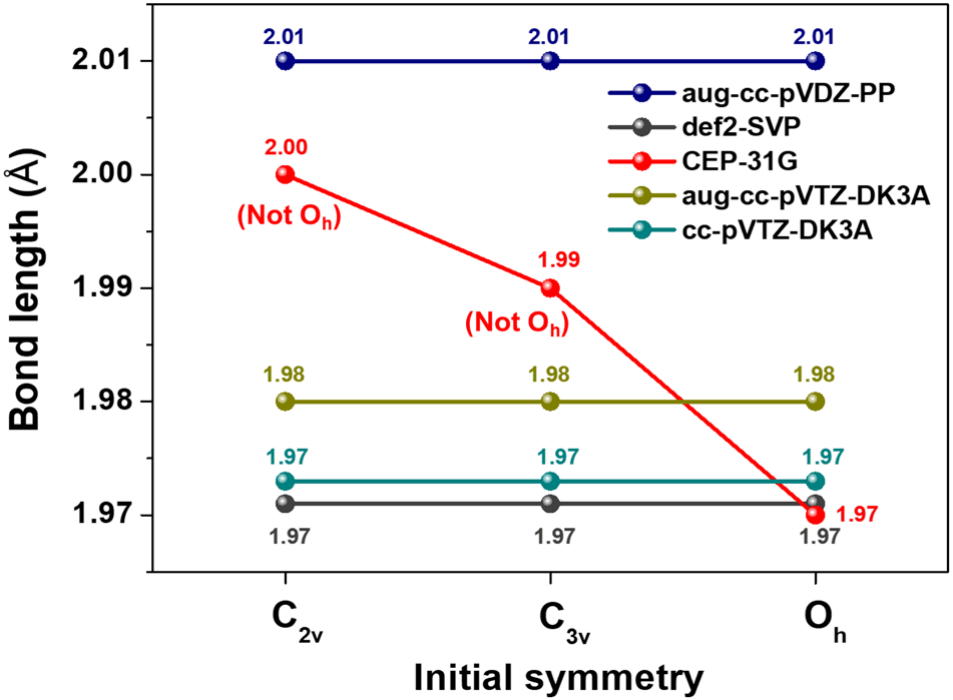
The bond lengths of RnF_6_ including all calculations by the CCSD method. *The calculated values^29^ with relativistic effect (Rel) and non-relativistic effect (NR).

Also, it is worth to note that the ground-state geometry of XeF_6_ is rather different from that of RnF_6_ when computed using the same methods and basis sets. To compare relativistic effects between Xe and Rn molecules, the molecular electrostatic potential (MEP) of RnF_6_ and XeF_6_ was mapped, as shown in Fig. 2. The molecular electrostatic potential surfaces describe the charge distribution of molecules in three dimensions^34^. Figure 2a shows a sigma hole (σ-hole), a region with a positive surface electrostatic potential^35^ in the MEP of XeF_6_(C_3v_). The lone pair of Xe experiences an electron shielding effect that shields a partial positive charge in the Xe nucleus, inducing a σ-hole due to the repulsive force between the Xe lone pair and the three F atoms^36^. In contrast, RnF_6_ has evenly distributed σ-holes in the middle of each face of the octahedral^37^, as shown in Fig. 2b. The bond lengths of RnF_6_ calculated using NR and DKH calculations and the CCSD method are shown in Fig. 3. Evidently, RnF_6_ converged equivalently to O_h_ symmetry for all basis sets, except for CEP-31G, even in the case of the DKH calculations.

### Vibrational spectra

Infrared (IR) vibrational spectra were obtained at the CCSD level, considering the relativistic effect, for the optimized structures of radon fluorides with different stoichiometries. The calculated vibrational spectra obtained with the aug-cc-pVTZ-DK3 basis set are tabulated in Table 4, wherein the calculated IR spectra are plotted against the wavenumbers. As no studies conducted thus far have reported the IR spectra of Rn molecules, these could not be compared with experimentally obtained frequencies. For RnF_6_, the all given initial structures converged to O_h_ symmetry, and therefore, a frequency comparison for each structure was meaningless. In the case of XeF_6_, the C_2v_ structure had one imaginary frequency, the O_h_ structure had a three-fold degenerate imaginary frequency, whereas Rn had no imaginary frequency. The presence of an imaginary frequency implies that the optimized structure under investigation is not stable^26^. This suggests that the optimized structures of radon fluoride were harmonically stable on the potential energy surface of the molecules. Note that the calculated frequencies at 648.28 cm^−1^, consistent with the e_u_ (double degeneracy) mode, accounted for most of the IR active frequency of RnF_4_. The calculated vibrational spectra for RnF_6_ indicated that the t_1u_ bending mode (at 663.93 cm^−1^) contributed to the greatest extent to the intensity. As RnF_4_ and RnF_6_ have not been characterized experimentally, our predicted vibrational spectra can play as a critical reference for future studies.

**Table 4:** IR spectroscopy with relativistic effect by CCSD of RnF_2_, RnF_4,_ and RnF_6_. The basis set of radon used in this calculation is aug-cc-pVTZ-DK3A.

Harmonic frequencies (cm^−1^)		
RnF_2_	RnF_4_	RnF_6_ (O_h_)
		136.64 (t_1u_)
		145.24 (t_2g_)
	155.73 (e_u_)	
	166.92 (b_2u_)	
195.64 (Bending)	224.71 (b_1g_)	224.87 (t_1u_)
	265.78 (a_2u_)	
585.33 (Symmetrical stretch)		
610.49 (Asymmetrical stretch)	609.55 (b_2g_)	
	631.67 (a_1g_)	631.33 (e_g_)
	648.27 (e_u_)	663.93 (t_1u_)
		678.72 (a_1g_)

### Dissociation energy

The dissociation energy of radon fluorides was predicted at CCSD levels with DKH calculations. Previous studies described the dissociation energies of xenon fluoride, which were obtained using classical thermodynamic equilibrium measurements with predicted entropies^38^. Likewise, this study described the thermodynamic reaction and dissociation energy of MF_6_ (M = Xe or Rn) using the following expressions.

1 $${\text{MF}}_{{6}} \, \to \,{\text{MF}}_{{4}} \, + \,{\text{F}}_{{2}} ,$$

2 $${\text{MF}}_{{6}} \, \to \,{\text{MF}}_{{2}} \, + \,{\text{2F}}_{{2}} ,$$

3 $${\text{MF}}_{{6}} \, \to \,{\text{M}}\, + \,{\text{3F}}_{{2}} .$$

Previous studies have described the heat of formation calculations for XeF_6_, and obtained – 62.1 ± 1.4 kcal/mol at 0 K and − 64.0 ± 1.4 kcal/mol at 298 K^26^. In this study, the dissociation energy of XeF_6_ (C_3v_) → Xe + 3F_2_ for the most stable structure, which was obtained consistently with the measured values, was 5.04 kcal/mol smaller than the experimentally obtained equilibrium value^38^, and 26.94 kcal/mol lower than that from the photoionization experiment^39^, as shown in Supplementary Fig. S2. Several previous studies reported errors in the experimental equilibrium values of 1–2 kcal/mol, which depended on structural differences^8^. However, the difference in the total dissociation energy in this study was relatively large, depending on symmetry. For example, the total dissociation energy of the CF_4_^+^ ion exhibited a variation of approximately 50 kcal/mol according to structural differences at the same level owing to the Jahn–teller effect^40^. Therefore, providing experimental evidence that addresses the question of energy differences according to XeF_6_ structures is challenging. The calculation results for XeF_6_ (C_3v_) using aug-cc-pVTZ-DK3A may be reasonable as these are rather similar to the equilibrium experimental values.

**Figure 4:**
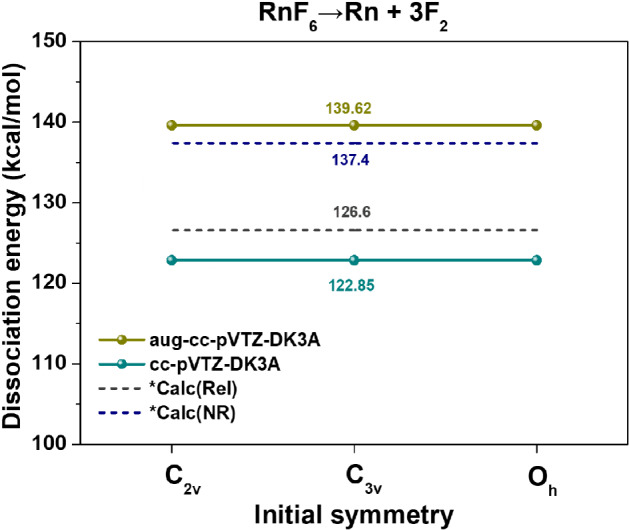
The dissociation energy of RnF_6_ → Rn + 3F_2_ with relativistic calculation by CCSD method. *The calculated values^16^ with relativistic effect (Rel) and non-relativistic effect (NR).

Dissociation energy was calculated according to various reactions of radon fluoride, and the results are presented in Supplementary Table S7; additionally, the dissociation energy of RnF_6_ → Rn + 3F_2_ is shown in Fig. 4. In general, the loss of F_2_ from RnF_2_ is slightly harder than the F_2_ loss from RnF_4_, and the loss of F_2_ in RnF_4_ is much harder than that in RnF_6_. This is consistent with the presence of a growing steric community as more fluoride is added to the central Rn^8^. Because convergence to O_h_ was achieved for all basis sets, no difference in the dissociation energy between Rn molecule structures was observed. There are no significant differences between previous studies^16^ with the MP2 method and our calculated values (aug-cc-pVTZ-DK3A: 139.62 kcal/mol and cc-pVTZ-DK3A: 122.85 kcal/mol, see Fig. 4).

## Discussion

Despite the necessity of studying the behavior of radioactive materials, such as Rn, which naturally occur in the environment, only radon difluoride (RnF_2_) and its complexes are known. Both a short half-life and radiological risks challenge the experimental assessment of Rn, and the existence of Rn molecules remains controversial. This study described the molecular geometry, vibrational spectra, and dissociation energies of the RnF_2_, RnF_4_, and RnF_6_ family of molecules using ab initio calculations.

Detailed parameters employed to describe xenon fluoride were used to obtain an optimized computational approach. For the XeF_6_ structure, the geometric parameters obtained at the CCSD level indicated a difference between calculated bond lengths of approximately 0.98% and angles that differed from other calculated values by only 1°. In addition, the dissociation energies for Xe + 3F_2_ were in good agreement with the experimental^38,39^ and other calculation references^26^. Using these calculations as a base, the geometry, dissociation energies, and vibrational spectra of RnF_2_, RnF_4_, and RnF_6_ were predicted.

CCSD-level calculations with relativistic effects provided radon fluoride bond lengths of 2.04 and 2.00 Å for radon di- and tetrafluoride, respectively. Unlike XeF_6_, which maintained C_3v_ symmetry, all possible initial group symmetries of RnF_6_ converged to an octahedral structure, with estimated bond lengths of approximately 1.97 Å. The dissociation energies for Rn and 3F_2_ were approximately 139.62 kcal/mol, which is higher than that of Xe. This may be assumed to be an accurate value, considering the high reactivity of Rn. This study can be extended by adding other anionic elements that can be possibly encountered in nature to the studied system, such as oxygen and chlorine, particularly in an aqueous environment. In addition, this study was the first to address the vibrational frequencies of radon di-, tetra-, and hexafluoride, and this information can be used as a basis for future radon-based experiments.

## Methods

### Computational methods

The initial molecular structures and formation energies of radon di-, tetra-, and hexafluoride were obtained via a*b initio* random structure searching (AIRSS)^41^ using the projected augmented plane-wave method (PAW) embedded in the Vienna ab initio simulation package (VASP)^42–44^. For structure searching, we placed a supercell with a volume of 10 × 10 × 10 Å^3^ and randomly generated Rn and F atoms for various Rn/F ratios. We searched ~ 5000 trial structures and 17 compositions. For formation energy calculations, the Perdew−Burke−Ernzerhof (GGA-PBE) exchange-correlation functional^45^ was used with a plane-wave basis set energy cutoff of 550 eV and the self-consistent-field energy convergence criteria of 1E−6 eV. Only a gamma-point k-mesh and a cubic unit cell with a lattice constant of 15 Å were used in this study.

All ab initio calculations for structural optimization and vibrational spectra were performed using the Gaussian09 simulation package^46^. The optimized molecular geometries of Xe and Rn fluorides were obtained using DFT and two different levels of the post Hartree–Fock (HF) theory method: MP2^21^, and the coupled-cluster (CC) method. In this study, the B3LYP hybrid function, which is one of the hybrid functionals provided by the combination of Becke’s three-parameter functional^47^ with the Lee, Yang, and Parr correlation functional^48^, was used for DFT calculations. In addition, MP2 was developed by Moller and Plesset^21^ to address HF theory in the case of many-electron systems. This approach was used in this study because it is known to correct minor deficiencies in the HF method and has been widely applied to molecular energy correlation. The CC method is one of the most prevalent post-HF methods and applies to sufficiently large molecules. A feature of the CC method is that, as opposed to other calculation methods, it can be systematically improved by including a higher excitation operator^49^.

The CCSD^22–25^ was used in our study, with higher excitations approximated by a product of lower excitations. Further, Gaussian09 was used at all levels in these calculations, with an SCF energy cutoff of 1E–6 Hartree.

### Nonrelativistic calculations (NR)

Three basis sets were used for Xe and Rn. For nonrelativistic calculations, all methods (DFT, MP2, and CCSD) were used with a variety of basis sets, namely LANL2DZ^50^, def2-SVP^51^, and CEP-31G^52–54^ for Xe. Furthermore, the aug-cc-pVDZ-PP^55^, def2-SVP^51^, and CEP-31G^52–54^ basis sets were defined for Rn. For comparison, using the same basis sets for both elements produced better results. However, LANL2DZ could not be used for radon because the largest atom was defined as bismuth (Bi).

### Relativistic calculations (DKH)

Relativistic effects were considered within the 2nd-order Douglas–Kroll–Hess (DKH) Hamiltonian implemented in the Gaussian09 software. Although this method does not involve spin–orbit coupling (SOC), the inclusion of SOC usually does not alter the molecular geometry qualitatively significantly^56^. The aug-cc-pVTZ-DK3 and cc-pVTZ-DK3 basis sets were selected for Xe and Rn, which is the only possible basis set both can be used for Xe and Rn. In addition, the effective core potential (ECP) was employed with both basis sets used to describe Rn. These extended ECP valence basis sets are denoted as “DK3A”. In each calculation that involved NR and DKH, the 6-31G++^57^ basis set with polarization and diffuse functions was used for fluorine in the molecules.

## Supplementary Information

Supplementary Information.

## Data Availability

The data that support the findings of this study are available from the corresponding author upon reasonable request.

## References

[1] Pauling L (1933). The formulas of antimonic acid and the antimonates. J. Am. Chem. Soc..

[2] Labinger J (2015). Why isn’t noble gas chemistry 30 years older? The failed (?) 1933 experiment of Yost and Kaye. Bull. Hist. Chem..

[3] Claassen HH, Selig H, Malm JG (1962). Xenon tetrafluoride. J. Am. Chem. Soc..

[4] Hyman HH (1964). The chemistry noble gas compounds. Science.

[5] Seppelt K (2003). Nonoctahedral structures. Acc. Chem. Res..

[6] Bartell LS, Gavin RM (1968). Molecular structure of XeF6. II. Internal motion and mean geometry deduced by electron diffraction. J. Chem. Phys..

[7] Claassen HH, Goodman GL, Kim H (1972). Spectral observations on molecular XeF6: Raman scattering and infrared, visible and ultraviolet absorption in the vapor and in matrix isolation. J. Chem. Phys..

[8] Dixon DA, de Jong WA, Peterson KA, Christe KO, Schrobilgen GJ (2005). Heats of formation of xenon fluorides and the fluxionality of XeF6 from high level electronic structure calculations. J. Am. Chem. Soc..

[9] Greenwood NN, Earnshaw A (2012). Chemistry of the Elements.

[10] Samet JM (1989). Radon and lung cancer. JNCI.

[11] Fields PR, Stein L, Zirin MH (1962). Radon fluoride. J. Am. Chem. Soc..

[12] Lee EPF, Wright TG (1999). Interaction energy of the radon-water (Rn, H2O) complex. J. Phys. Chem. A.

[13] Kang J, Singh BK, Um W (2021). Efficient radon removal using fluorine-functionalized natural zeolite. J. Environ. Radioact..

[14] Liao M-S, Zhang Q-E (1998). Chemical bonding in XeF2, XeF4, KrF2, KrF4, RnF2, XeCl2, and XeBr 2: From the gas phase to the solid state. J. Phys. Chem. A.

[15] Malli GL (2001). Relativistic all-electron Dirac-Fock calculations on RnF6 and its ions. J. Mol. Struct. (Thoechem.).

[16] Filatov M, Cremer D (2003). Bonding in radon hexafluoride: An unusual relativistic problem?. Phys. Chem. Chem. Phys..

[17] Gillespie RJ, Hargittai I (1991). The VSEPR Model of Molecular Geometry.

[18] Hedberg K, Peterson SH, Ryan RR, Weinstock B (1966). On the structure of gaseous XeF_6_. J. Chem. Phys..

[19] Pitzer KS, Bernstein LS (1975). Molecular structure of XeF6. J. Chem. Phys..

[20] Kaupp M, van Wüllen C, Franke R, Schmitz F, Kutzelnigg W (1996). The structure of XeF6 and of compounds isoelectronic with it. A challenge to computational chemistry and to the qualitative theory of the chemical bond. J. Am. Chem. Soc..

[21] Møller C, Plesset MS (1934). Note on an approximation treatment for many-electron systems. Phys. Rev..

[22] Purvis GD, Bartlett RJ (1982). A full coupled-cluster singles and doubles model: The inclusion of disconnected triples. J. Chem. Phys..

[23] Scuseria GE, Janssen CL, Schaefer HF (1988). An efficient reformulation of the closed-shell coupled cluster single and double excitation (CCSD) equations. J. Chem. Phys..

[24] Scuseria GE, Schaefer HF (1989). Is coupled cluster singles and doubles (CCSD) more computationally intensive than quadratic configuration interaction (QCISD)?. J. Chem. Phys..

[25] Cížek J (1969). Advances in Chemical Physics.

[26] Peterson KA, Dixon DA, Stoll H (2012). The use of explicitly correlated methods on XeF6 predicts a C3v minimum with a sterically active, free valence electron pair on Xe. J. Phys. Chem. A.

[27] Cheng L, Gauss J, Stanton JF (2015). Relativistic coupled-cluster calculations on XeF6: Delicate interplay between electron-correlation and basis-set effects. J. Chem. Phys..

[28] Desclaux JP, Kim Y-K (1975). Relativistic effects in outer shells of heavy atoms. J. Phys. B.

[29] Wilson AK (2018). Heaviest element has unusual shell structure. Physics.

[30] Braïda B, Hiberty PC (2013). The essential role of charge-shift bonding in hypervalent prototype XeF2. Nat. Chem..

[31] Avrorin VV, Krasikova RN, Nefedov VD, Toropova MA (1982). The chemistry of radon. Russ. Chem. Rev..

[32] Han Y-K, Lee YS (1999). Structures of RgF n (Rg= Xe, Rn, and element 118. n= 2, 4.) calculated by two-component spin-orbit methods. A spin-orbit induced isomer of (118) F4. J. Phys. Chem. A.

[33] Grandinetti F (2018). Noble Gas Chemistry: Structure, Bonding, and Gas-Phase Chemistry.

[34] Lakshminarayanan S, Jeyasingh V, Murugesan K, Selvapalam N, Dass G (2021). Molecular electrostatic potential (MEP) surface analysis of chemo sensors: An extra supporting hand for strength, selectivity & non-traditional interactions. J. Photochem. Photobiol..

[35] Wang H, Wang W, Jin WJ (2016). σ-hole bond vs π-hole bond: A comparison based on halogen bond. Chem. Rev..

[36] Haner J, Matsumoto K, Mercier HPA, Schrobilgen GJ (2016). Nature of the XeVI−N bonds in F6XeNCCH3 and F6Xe(NCCH3)2 and the stereochemical activity of their xenon valence electron lone pairs. Chemistry.

[37] Frontera A (2020). Noble gas bonding interactions involving xenon oxides and fluorides. Molecules.

[38] Weinstock B, Weaver EE, Knop CP (1966). The xenon-fluorine system. Inorg. Chem..

[39] Woolf AA (1981). Advances in Inorganic Chemistry and Radiochemistry.

[40] de la Vega JMG, Fabián ES (1991). Jahn-Teller effect and dissociation from the ground state of CF4+. Chem. Phys..

[41] Pickard CJ, Needs R (2006). High-pressure phases of silane. Phys. Rev. Lett..

[42] Kresse G, Hafner J (1994). Ab initio molecular-dynamics simulation of the liquid-metal–amorphous-semiconductor transition in germanium. Phys. Rev. B.

[43] Kresse G, Furthmüller J (1996). Efficiency of ab-initio total energy calculations for metals and semiconductors using a plane-wave basis set. Comput. Mater. Sci..

[44] Kresse G, Furthmüller J (1996). Efficient iterative schemes for ab initio total-energy calculations using a plane-wave basis set. Phys. Rev. B.

[45] Perdew JP, Burke K, Ernzerhof M (1996). Generalized gradient approximation made simple. Phys. Rev. Lett..

[46] Gaussian09, R. *et al.* Gaussian 09, Revision E. 01, Gaussian, Inc, Wallingford, CT, 2004. *Inc., Wallingford CT* **121**, 150–166 (2009).

[47] Becke AD (1993). Density-functional thermochemistry. III. The role of exact exchange. J. Chem. Phys..

[48] Calhorda MJ, Pregosin PS, Veiros LF (2007). Geometry optimization of a Ru(IV) allyl dicationic complex: A DFT failure?. J. Chem. Theory Comput..

[49] Bartlett RJ, Musiał M (2007). Coupled-cluster theory in quantum chemistry. Rev. Mod. Phys..

[50] Wadt WR, Hay PJ (1985). Ab initio effective core potentials for molecular calculations. Potentials for main group elements Na to Bi. J. Chem. Phys..

[51] Weigend F, Ahlrichs R (2005). Balanced basis sets of split valence, triple zeta valence and quadruple zeta valence quality for H to Rn: Design and assessment of accuracy. Phys. Chem. Chem. Phys..

[52] Stevens WJ, Basch H, Krauss M (1984). Compact effective potentials and efficient shared-exponent basis sets for the first-and second-row atoms. J. Chem. Phys..

[53] Stevens WJ, Krauss M, Basch H, Jasien PG (1992). Relativistic compact effective potentials and efficient, shared-exponent basis sets for the third-, fourth-, and fifth-row atoms. Can. J. Chem..

[54] Cundari TR, Stevens WJ (1993). Effective core potential methods for the lanthanides. J. Chem. Phys..

[55] Peterson KA (2003). Systematically convergent basis sets with relativistic pseudopotentials. I. Correlation consistent basis sets for the post-d group 13–15 elements. J. Chem. Phys..

[56] Xiao L, Wang L (2004). From planar to three-dimensional structural transition in gold clusters and the spin–orbit coupling effect. Chem. Phys. Lett..

[57] Clark T, Chandrasekhar J, Spitznagel GW, Schleyer PVR (1983). Efficient diffuse function-augmented basis sets for anion calculations. III. The 3–21+G basis set for first-row elements, Li–F. J. Comput. Chem..

