# Supplementary material for: Prediction of stable radon fluoride molecules and geometry optimization using first-principles calculations
Source: Sci Rep. 2023 Feb 18;13:2898. doi: 10.1038/s41598-023-29313-5 (PMC9938903; doi:10.1038/s41598-023-29313-5)
Supplement: Supplementary file 1 — Supplementary Information. [file 41598_2023_29313_MOESM1_ESM.docx]

**Supplementary Information**

Prediction of stable radon fluoride molecules and geometry optimization using first-principles calculations

Jaeeun Kang^1^, Ina Park^2^, Ji Hoon Shim^2^, Duck Young Kim^2, 3, ∗^, and Wooyong Um^1, 4, 5, ∗^

^1^Division of Advanced Nuclear Engineering (DANE), Pohang University of Science and Technology (POSTECH), 77 Cheongam–ro, Nam–Gu, Pohang, Gyeongbuk, 790–784, Republic of Korea

^2^Department of Chemistry, Pohang University of Science and Technology (POSTECH), 77 Cheongam–ro, Nam–Gu, Pohang, Gyeongbuk, 790–784, Republic of Korea

^3^Center for High Pressure Science & Technology Advanced Research, Shanghai, People’s Republic of China

^4^Division of Environmental Sciences and Engineering (DESE), Pohang University of Science and Technology (POSTECH), 77 Chongam–ro, Nam–Gu, Pohang 790–784, Republic of Korea

^5^Nuclear Environmental Technology Institute (NETI), Pohang University of Science and Technology (POSTECH), Pohang, Gyeongbuk 790–784, Republic of Korea

*Correspondence: duckyoung.kim@hpstar.ac.cn, [wooyongum@postech.ac.kr](mailto:wooyongum@postech.ac.kr)

**Supplementary Table S1.** Geometric parameters were determined by DFT for the C_2v_, C_3v_, and O_h_ structures of XeF_6_.

| Basis set | Initial  symmetry | Bond length (Å) | Bond angle (º) | Energy (Hartree) | ΔE  (kcal/mol) |
| --- | --- | --- | --- | --- | --- |
| LANL2DZ | C_2v_ | a = 1.99  b =1.94  c =1.90 | α =110.17  β = 81.23 | – 613.91 | – 1.19 |
|  | C_3v_ | a = 1.98  b =1.89 | α = 109.02  β = 81.12 | – 613.91 |  |
|  | O_h_ | r = 1.95 | θ = 90 | – 613.91 | – 2.07 |
| def2-SVP | ^*^C_2v_ | a, b, c = 1.95 | α, β = 90 | – 928.13 | – 0.19 |
|  | ^*^C_3v_ | a, b = 1.95 | α, β = 90 | – 928.13 |  |
|  | O_h_ | r = 1.95 | θ = 90 | – 928.13 | 0.00 |
| CEP-31G | C_2v_ | a = 1.99  b =1.94  c =1.90 | α = 103.92  β = 83.18 | – 613.97 | – 0.56 |
|  | C_3v_ | a = 2.00  b =1.92 | α = 107.91  β = 81.68 | – 613.97 |  |
|  | O_h_ | r = 1.98 | θ = 90 | – 613.97 | – 0.75 |
| ^**^Experiment^19^ | | a = 1.94  b =1.85 | α = 114.90  β = 81.0 |  | |

^*^ Initial symmetry converged equivalently to O_h_. ^**^ The bond angles are the results of all-electron calculations at the SCF level.

Supplementary Table S2. Geometric parameters were determined by MP2 for the C_2v_, C_3v_, and O_h_ structures of XeF_6_.

| Basis set | Initial  symmetry | Bond length (Å) | Bond angle (º) | Energy (Hartree) | ΔE  (kcal/mol) |
| --- | --- | --- | --- | --- | --- |
| LANL2DZ | ^*^C_2v_ | a, b, c = 1.94 | α, β = 90 | – 611.05 | – 30.48 |
|  | C_3v_ | a = 1.95  b =1.85 | α = 111.61  β = 80.96 | – 611.10 |  |
|  | O_h_ | r = 1.94 | θ = 90 | – 611.05 | – 30.60 |
| def2-SVP | ^*^C_2v_ | a, b, c = 1.95 | α, β = 90 | – 924.30 | 0.06 |
|  | ^*^C_3v_ | a, b = 1.95 | α, β = 90 |  |  |
|  | O_h_ | r = 1.95 | θ = 90 |  | 0.06 |
| CEP-31G | ^*^C_2v_ | a = 1.99  b =1.94  c =1.91 | α, β = 90 | – 611.09 | – 26.78 |
|  | C_3v_ | a = 2.00  b =1.92 | α = 109.79  β = 80.71 | – 611.13 |  |
|  | O_h_ | r = 1.98 | θ = 90 | – 611.09 | – 26.84 |
| ^**^Experiment^19^ | | a = 1.94  b =1.85 | α = 114.90  β = 81.0 |  | |

^*^ Initial symmetry converged equivalently to O_h_. ^**^ The bond angles are the results of all-electron calculations at the SCF level.

**Supplementary Table S3.** Geometric parameters were determined by CCSD for the C_2v_, C_3v_, and O_h_ structures of XeF_6_.

| Basis set | Initial  symmetry | Bond length (Å) | Bond angle (º) | Energy (Hartree) | ΔE  (kcal/mol) |
| --- | --- | --- | --- | --- | --- |
| LANL2DZ | C_2v_ | a = 1.96  b =1.88  c =1.85 | α = 128.25  β = 80.06 | – 611.10 | – 6.59 |
|  | C_3v_ | a = 1.94  b =1.83 | α = 113.11  β = 80.178 | – 611.11 |  |
|  | O_h_ | r = 1.94 | θ = 90 | – 611.06 | – 30.54 |
| def2-SVP | C_2v_ | a = 1.99  b =1.90  c = 1.86 | α = 121.64  β = 84.32 | – 924.33 | – 2.57 |
|  | C_3v_ | a = 1.97  b =1.86 | α = 108.63  β = 82.40 | – 924.34 |  |
|  | O_h_ | r = 1.95 | θ = 90 | – 924.31 | – 17.62 |
| CEP-31G | C_2v_ | a = 1.98  b =1.90  c =1.86 | α = 129.08  β = 82.21 | – 611.13 | – 6.27 |
|  | C_3v_ | a = 1.96  b =1.86 | α = 113.15  β = 80.30 | – 611.14 |  |
|  | O_h_ | r = 1.96 | θ = 90 | – 611.09 | – 29.85 |
| ^**^Experiment^19^ | | a = 1.94  b =1.85 | α = 114.90  β = 81.0 |  | |

^**^ The bond angles are the results of all-electron calculations at the SCF level.

**Supplementary Table S4.** Geometric parameters were determined with relativistic effect (DKH calculation) by CCSD for the C_2v_, C_3v_, and O_h_ structures of XeF_6_.

| Basis set | Initial  symmetry | Bond length (Å) | Bond angle (º) | Energy  (Hartree) | ΔE  (kcal/mol) |
| --- | --- | --- | --- | --- | --- |
| aug-cc-pVTZ-DK3 | C_2v_ | a = 1.98  b =1.90  c =1.87 | α = 124.76  β = 83.94 | – 7547.27 | – 10.41 |
|  | C_3v_ | a = 1.97  b = 1.87 | α = 109.62  β = 81.87 | – 7547.28 |  |
|  | O_h_ | r = 1.98 | θ = 90 | – 7547.18 | – 66.98 |
| cc-pVTZ-DK3 | C_2v_ | a = 1.97  b =1.90  c =1.87 | α = 124.19  β = 84.08 | – 7547.18 | – 8.28 |
|  | C_3v_ | a = 1.96  b = 1.86 | α = 109.46  β = 81.97 | – 7547.19 |  |
|  | O_h_ | r = 1.93 | θ = 90º | – 7547.16 | – 18.88 |
| ^**^Experiment^19^ | | a = 1.94  b =1.85 | α = 114.90  β = 81.0 |  | |

^**^ The bond angles are the results of all-electron calculations at the SCF level.

**Supplementary Table S5.** Geometric parameters were determined by DFT for the C_2v_, C_3v_, and O_h_ structures of RnF_6_. All basis sets were converged equivalently to O_h_.

| Basis set | Initial  symmetry | Bond length (Å) | Energy (Hartree) | Converged symmetry |
| --- | --- | --- | --- | --- |
| aug-cc-pVDZ | C_2v_ | a, b, c = 2.03 | – 887.44 | O_h_ |
|  | C_3v_ | a, b = 2.03 |  |  |
|  | O_h_ | r = 2.03 |  |  |
| def2-SVP | C_2v_ | a, b, c = 1.99 | – 887.49 | O_h_ |
|  | C_3v_ | a, b = 1.99 |  |  |
|  | O_h_ | r = 1.99 |  |  |
| CEP-31G | C_2v_ | a = 2.01  b, c =2.00 | – 613.51 | O_h_ |
|  | C_3v_ | a, b = 2.01 |  |  |
|  | O_h_ | r = 2.01 |  |  |

**Supplementary Table S6.** Geometric parameters were determined by MP2 for the C_2v_, C_3v,_ and O_h_ structures of RnF_6_. All basis sets were converged equivalently to O_h_.

| Basis set | Initial  symmetry | Bond length (Å) | Energy (Hartree) | Converged symmetry |
| --- | --- | --- | --- | --- |
| aug-cc-pVDZ | C_2v_ | a, b, c = 2.03 | – 883.58 | O_h_ |
|  | C_3v_ | a, b = 2.03 |  |  |
|  | O_h_ | r = 2.03 |  |  |
| def2–SVP | C_2v_ | a, b, c = 1.98 | – 883.65 | O_h_ |
|  | C_3v_ | a, b = 1.98 |  |  |
|  | O_h_ | r = 1.98 |  |  |
| CEP–31G | C_2v_ | a, b, c = 1.99 | – 610.67 | O_h_ |
|  | C_3v_ | a, b = 1.99 |  |  |
|  | O_h_ | r = 1.99 |  |  |

**Supplementary Table S7.** The dissociation energies were determined with relativistic effect by CCSD of RnF_2_, RnF_4,_ and RnF_6_.

| Molecules | Reaction | Basis set | Energy (kcal/mol) |
| --- | --- | --- | --- |
| RnF_2_ | RnF_2_ → Rn + F_2_ | aug-cc-pVTZ-DK3A | 39.09 |
|  |  | cc-pVTZ-DK3A | 29.87 |
| RnF_4_ | RnF_4_ → Rn + 2F_2_ | aug-cc-pVTZ-DK3A | 86.62 |
|  |  | cc-pVTZ-DK3A | 71.70 |
|  | RnF_4_ → RnF_2_ + F_2_ | aug-cc-pVTZ-DK3A | 47.53 |
|  |  | cc-pVTZ-DK3A | 41.84 |
| RnF_6_ | RnF_6_ → Rn + 3F_2_ | aug-cc-pVTZ-DK3A | 139.62 |
|  |  | cc-pVTZ-DK3A | 122.85 |
|  | RnF_6_ → RnF_4_ + F_2_ | aug-cc-pVTZ-DK3A | 53.01 |
|  |  | cc-pVTZ-DK3A | 51.15 |
|  | RnF_6_ → RnF_2_ + 2F_2_ | aug-cc-pVTZ-DK3A | 100.54 |
|  |  | cc-pVTZ-DK3A | 92.99 |


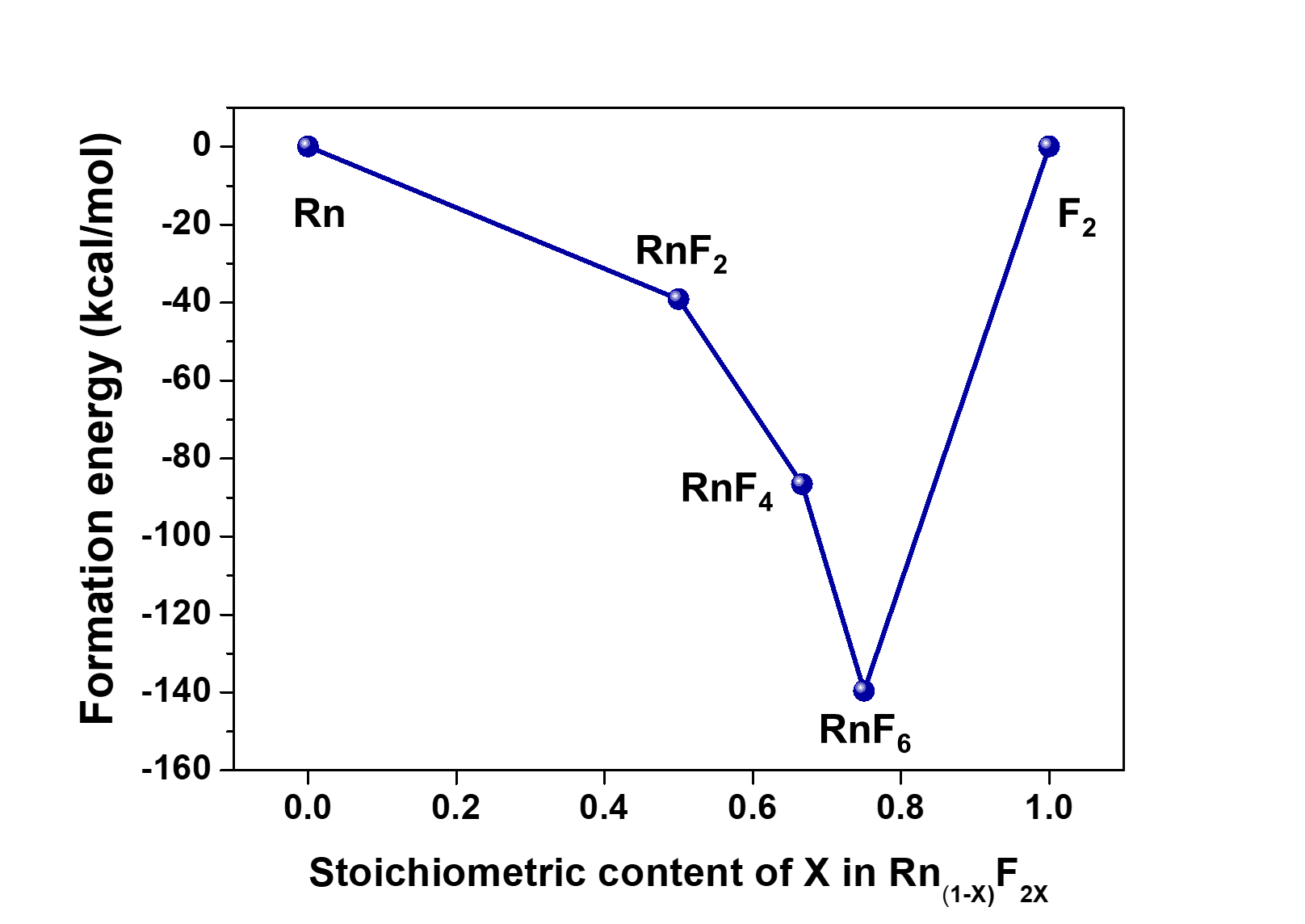


**Supplementary Figure S1.** Convex hull graph of radon fluorides as well as Rn and F_2_ with formation energy (kcal/mol).


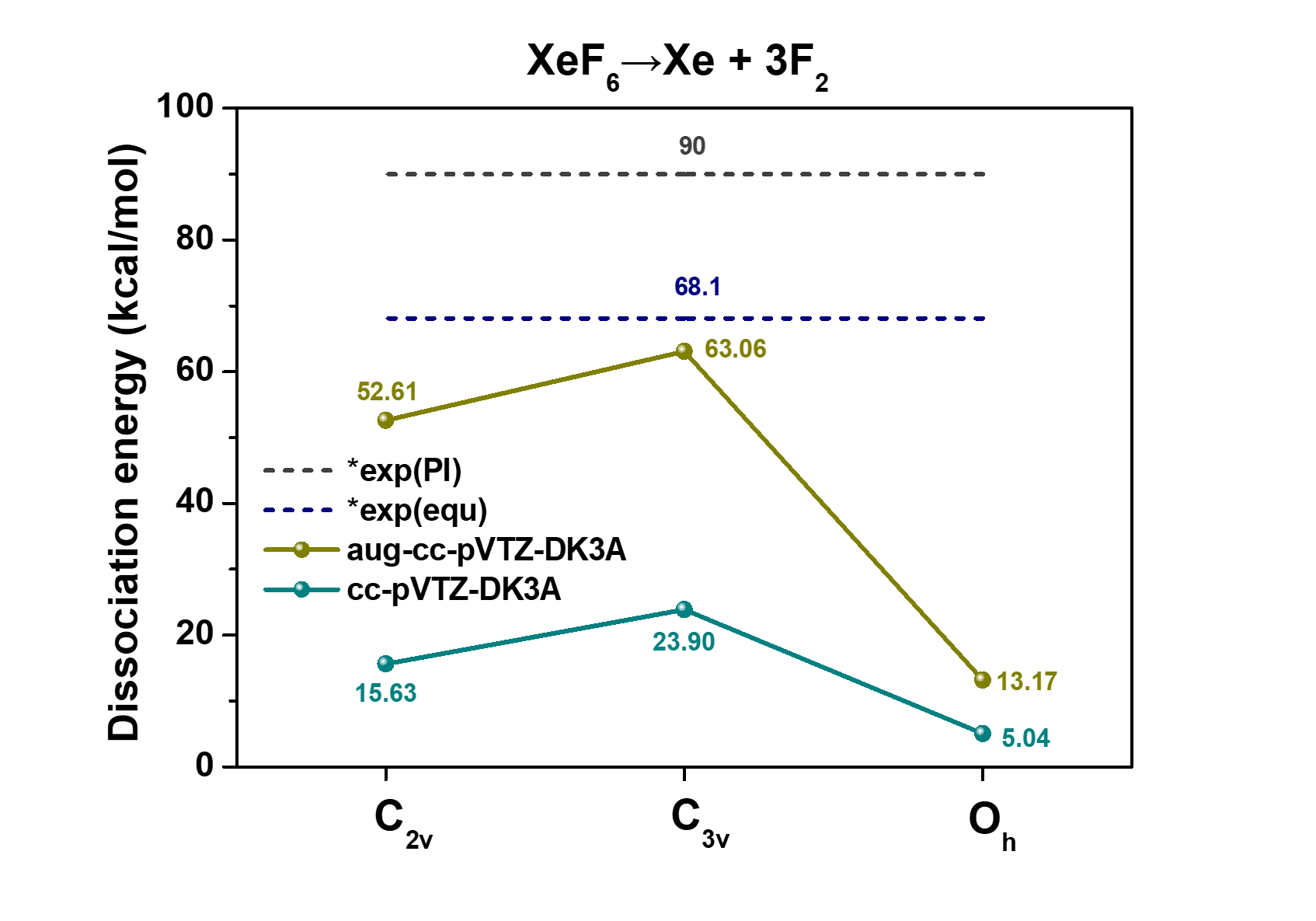


**Supplementary Figure S2.** The dissociation energy of XeF_6_ → Xe +3F_2_ with DKH calculation by CCSD method. The values obtained from the equilibrium^38^ (equ) and photoionization^39^ (PI) experiments.
